# Shared Bacterial and Viral Respiratory Agents in Bighorn Sheep (*Ovis canadensis*), Domestic Sheep (*Ovis aries*), and Goats (*Capra hircus*) in Montana

**DOI:** 10.4061/2011/162520

**Published:** 2011-11-16

**Authors:** David S. Miller, Glen C. Weiser, Keith Aune, Brent Roeder, Mark Atkinson, Neil Anderson, Thomas J. Roffe, Kim A. Keating, Phillip L. Chapman, Cleon Kimberling, Jack Rhyan, P. Ryan Clarke

**Affiliations:** ^1^Department of Clinical Sciences, College of Veterinary Medicine and Biomedical Sciences, Colorado State University, Fort Collins, CO 80523, USA; ^2^University of Idaho, College of Agricultural and Life Sciences, Caine Veterinary Teaching Center, 1020 East Homedale Road, Caldwell, ID 83607, USA; ^3^Montana Fish, Wildlife & Parks, 1400 South 19th Avenue, Bozeman, MT 59715, USA; ^4^Wildlife Conservation Society, 2023 Stadium Drive, Suite 1A, Bozeman, MT 59715, USA; ^5^Department of Animal & Range Sciences, Montana State University, P.O. Box 172900, Bozeman, MT 59717, USA; ^6^Wildlife Conservation Society, 2300 Southern Boulevard, Bronx, NY 10460, USA; ^7^U.S. Fish and Wildlife Service, 1400 S 19th Avenue, Bozeman, MT 59718, USA; ^8^U.S. Geological Survey, Northern Rocky Mountain Science Center, 2327 University Way, Suite 2, Bozeman, MT 59715, USA; ^9^Department of Statistics, Colorado State University, 224 Statistics Building, Fort Collins, CO 80523, USA; ^10^National Wildlife Research Center, 4101 La Porte Avenue, Fort Collins, CO 80521, USA; ^11^Animal and Plant Health Inspection Service, U.S. Department of Agriculture, Western Region, Belgrade, MT 59714, USA

## Abstract

Transmission of infectious agents from livestock reservoirs has been hypothesized to cause respiratory disease outbreaks in bighorn sheep (*Ovis canadensis*), and land management policies intended to limit this transmission have proven controversial. This cross-sectional study compares the infectious agents present in multiple populations of bighorn sheep near to and distant from their interface with domestic sheep (*O. aries*) and domestic goat (*Capra hircus*) and provides critical baseline information needed for interpretations of cross-species transmission risks. Bighorn sheep and livestock shared exposure to Pasteurellaceae, viral, and endoparasite agents. In contrast, although the impact is uncertain, *Mycoplasma* sp. was isolated from livestock but not bighorn sheep. These results may be the result of historic cross-species transmission of agents that has resulted in a mosaic of endemic and exotic agents. Future work using longitudinal and multiple population comparisons is needed to rigorously establish the risk of outbreaks from cross-species transmission of infectious agents.

## 1. Introduction

Bighorn sheep (*Ovis canadensis*) experienced substantial decreases in population numbers and range in the 19th and the early 20th centuries, and subsequent recovery efforts have often been limited by large-scale die-offs [1–3]. These initial population declines were associated with settlement of western North America and were attributed to unregulated hunting, competition for forage with domestic sheep (*O. aries*) and other livestock, and disruption of historic bighorn sheep migration patterns due to development. Clinical disease was apparently unimportant or was underreported in these early declines, though die-offs of bighorn sheep associated with sheep scab (*Psoroptes* sp.) were reported following settlement [4, 5].

Bighorn sheep die-offs associated with pneumonia were reported in the 1920s and 1930s [6–10]. These early reports and subsequent work largely focused on lungworm (*Protostrongylus* sp.) as the primary infectious agent, although the involvement of *Pasteurella* sp., *Corynebacterium pyogenes* (currently *Arcanobacterium pyogenes*), and other host and environmental determinants were also noted as potential causes of respiratory disease. Subsequently, inconsistent association of lungworm with respiratory disease in bighorn sheep, as well as further evidence for *Pasteurella* sp. as the cause of pneumonia, led to a focus on pasteurellosis as a cause of respiratory disease outbreaks [11–14]. This research included evidence that *Pasteurella* sp. strains from clinically normal domestic sheep were pathogenic to bighorn sheep, and a molecular basis for this observation was established [15–17]. Much of this research was conducted under captive conditions or in vitro, due to the challenges of identifying morbid or recently dead animals that are appropriate for sampling, variation in methods for investigating outbreaks, and other challenges for conducting field investigations on bighorn sheep diseases. Recent evidence confirms that transmission of *Mannheimia haemolytica* (formerly *Pasteurella haemolytica*) from domestic sheep to bighorn sheep can occur under experimental conditions when comingling [18]. Alternative hypotheses that are not mutually exclusive with pasteurellosis include other infectious agents, external stressors, and nutritional deficiencies that can lead to compromised bighorn sheep immunity to infectious disease [19–21]. An understanding of the etiopathogenesis of bighorn sheep respiratory disease outbreaks is further complicated by inconsistent isolation of agents such as *Mycoplasma ovipneumoniae* and viruses from bighorns during such outbreaks [22, 23]. In addition, pneumonia in lambs has been described as a distinct phenomenon from that of adults [9, 24]. Understanding the complex etiopathogenesis of bighorn sheep bronchopneumonia in adult and juvenile bighorn sheep may provide managers with options to mitigate, halt, or prevent some bighorn sheep die-offs. 

The first step to better understand the complex relationships in the etiopathogenesis of bronchopneumonia is to identify the normal suite of viruses, bacteria, and parasites in healthy bighorn sheep and livestock populations. This report provides baseline data for Pasteurellaceae biovariants, *Mycoplasma* sp., selected viral respiratory agents, and endoparasites from multiple populations of apparently healthy bighorn and domestic sheep.

## 2. Materials and Methods

### 2.1. Populations Studied

Bighorn sheep populations were sampled opportunistically when management or research objectives permitted. Domestic sheep populations were sampled based on producer permission and whether located distant from or near known bighorn sheep but were not meant to be all inclusive for all populations at a given location. Four different types of populations were sampled using consistent methods: isolated and interface bighorn sheep populations and isolated and interface domestic sheep populations (Table 1). Isolated and interface populations were classified based on the location where the population was sampled, without inference as to historic movement of individuals or populations. Isolated domestic sheep populations consisted of populations that had one of the following characteristics: (a) bighorn sheep were not known to be within 14.5 km, or (b) bighorn sheep were prevented from commingling with domestics by physical barriers (housing development), (c) bighorn sheep were temporally separated from domestics by season of occupation. Bighorn sheep were reported by producers to be in pastures or within visual contact of pastures for each interface livestock population studied. For each interface bighorn sheep population, two nearby domestic sheep populations were identified for the purpose of evaluating shared virus, bacteria, and parasitic agents. One goat population that was comanaged with an interface domestic sheep population was also included in the study, due to the potential for transmission of agents between these species. The number of animals sampled in each population varied due to availability, and cost constraints limited testing of all animals for all agents. The threshold of 14.5 km distance was initially used for classifying “isolated” populations based on management guidelines for bighorn and domestic sheep, although subsequently superseded by visual observations of pasture contact [25]. Consequently, interface populations had pasture or closer contact, whereas isolated populations were those where pasture or closer contact was considered unlikely.

Isolated bighorn sheep populations included those located in or near Thompson Falls, Perma/Plains, Sun River, Charles M. Russell National Wildlife Refuge, National Bison Range, Glacier National Park, and Harper's Ferry (Montana Fish Wildlife and Parks (MFWP) population HD622), Montana [26]. Interface bighorn sheep populations included those near Winifred, Anaconda, and Helena, Montana. Domestic sheep population identification was coded due to participant confidentiality concerns. Locations for populations were recorded in World Geodetic System (WGS) 84 GPS format.

Bighorn sheep populations were characterized from winter aerial surveys conducted by MFWP in 2003. Domestic livestock populations were characterized from questionnaires verbally administered to the producers after sampling their flock.

### 2.2. Animal Handling and Sampling

Animal handling research protocols were approved by Institutional Animal Care and Use Committees at Colorado State University (protocol number ACUC 05-05-283A-01) and the US Geologic Survey's Northern Rocky Mountain Science Center (unnumbered protocol for work conducted in Glacier National Park). Bighorn sheep were captured in 2002–2006 during the months of September–June, as a part of routine research and management activities, and were conducted using a combination of physical (helicopter net gun) and chemical restraint techniques [27]. Domestic livestock were manually restrained during the spring or fall-2005-2006. Physical examination and biomedical sample collection procedures were comparable for all animals and were conducted as quickly as possible to minimize overheating and capture stress. Snow, water, or ethanol was applied to bighorn sheep to correct hyperthermia, as needed. Evidence of respiratory disease was noted, including nasal discharge or coughing. Uniquely identified individuals were resampled in three isolated domestic sheep populations and one goat population on two occasions, 6 mo apart, to determine temporal variability. All samples were uniquely identified by population, date, and individual.

Pasteurellaceae biovariants and subspecies (no. of isolates) cultured from individual isolated and interface bighorn sheep (*n* = 10 populations), domestic sheep (*n* = 12 populations), and goats (*n* = 1 population).

Sampling of the oropharynx of all animals for bacteria was conducted by opening the oral cavity with a mouth gag cleaned in soapy water and disinfected in 70% ethanol or by hands covered with fresh, disposable gloves per standardized protocols developed by the Western Wildlife Health Committee, Association of Western Fish and Wildlife Agencies [28]. A Dacron swab was used to sample the surface of the palatine tonsils and surrounding oropharyngeal region using methods developed for bighorn sheep [28]. The swab was immediately placed in sterile media tubes containing modified Cary Blair media (Port-a-cul, Becton-Dickinson, Franklin Lakes, NJ, USA) and placed on cold packs. The swab was shipped chilled without freezing to a reference laboratory (Caine Veterinary Teaching Center, University of Idaho, Caldwell, ID, USA) (CVTC) for *Pasteurellaceae* sp. and *Mycoplasma *sp. culture within 72 hr of collection. Swabs that contacted the tongue, teeth, or other potential sites of contamination were discarded, and the process was repeated until a sample representative of the oropharyngeal flora was collected. 

Blood was collected via jugular venipuncture into sterile serum collection tubes (Vacutainer, Becton-Dickinson, Franklin Lakes, NJ, USA). Blood samples were kept cool in the field by using cold packs. Samples were subsequently centrifuged the day of collection, and serum was removed. Serum was hand carried on cool packs or shipped frozen serum to the Montana Veterinary Diagnostic Laboratory (Bozeman, MT, USA) (MVDL) for viral serology. 

Feces were collected from the rectum or upon defecation during processing. Samples were kept chilled and submitted to the veterinary diagnostic laboratory for fecal floatation and Baermann analyses [29, 30]. 

Following Montana Fish Wildlife and Parks policy, a male bighorn sheep found to be commingling with domestic sheep and goats in animal shelters for more than six months was euthanized using approved procedures [31]. An oral swab and lung tissue were collected from this individual for Pasteurellaceae and *Mycoplasma* culture. Oral swabs were collected from commingling domestic sheep (*n* = 29) and domestic goats (*n* = 34).

### 2.3. Bacterial Culture and Classification Procedures

Consistent culture methods were used throughout the study over time and between samples sources. At CVTC, the oropharyngeal swab from each animal was inoculated onto nonselective Columbia blood agar (CBA) (Becton Dickinson & Co., Sparks, MD, USA), containing 5% sheep blood, and CBA with selective antibiotics, containing 5% bovine blood [32], and incubated for 24 hr at 37°C in a 10% CO_2_ atmosphere. Following incubation, representatives of each colony type were propagated on fresh CBA for species and Pasteurellaceae biovariant classification using previously described methods that are useful for wildlife studies [33, 34]. For the purposes of this report, each distinct bacteria that was identified among cultures from an oropharyngeal swab is called an isolate, even if multiple colonies of that bacteria were cultured from a single swab.

At CVTC, the swab was subsequently placed in *Mycoplasma* enrichment broth [35] and incubated at 37°C for 72 hr using methods that have previously identified *M. ovipneumoniae* in bighorn sheep [36]. Broth was subsequently streaked on *Mycoplasma* plates and incubated at 37°C with 5–10% CO_2_ for 5–7 days. Finally, *Mycoplasma* colonies were selected and plated on fresh medium. Not every sample was processed for *Mycoplasma*, but every population had a minimum of five animals cultured for *Mycoplasma*.

### 2.4. Serology Procedures

Serology was conducted at MVDL for antibodies to viruses with the potential to cause or predispose animals to respiratory infection. The serum neutralization (SN) test was used for infectious bovine rhinotracheitis (IBR) virus, bovine viral diarrhea virus (BVDV-1 and BVDV-2), and bovine respiratory syncytial virus (BRSV) [37–39] and hemagglutination inhibition for parainfluenza-3 (PI-3) [38, 40]. These methods classified serology titer results ≥ 1 : 8 as positive for antibodies to IBR, BVDV-1, BVDV-2, BRSV, and PI-3. Seroconversion was defined as a ≥ fourfold increase in titer for any of the four viruses.

### 2.5. Fecal Parasitology

Veterinary Parasitology Services used conventional fecal floatation to recover oocysts and eggs of gastrointestinal nematodes, cestodes, and protozoa, whereas Baermann assay methods were used to separate first-stage larvae of lungworms prior to identification [30]. Parasites were qualitatively reported as present or absent, without quantification. Eggs of Trichostongylina were not differentiated by genus. Larvae of Protostrongylidae and Dictyocaulidae strongylid larvae were identified to genus. Cost constraints prevented conducting assays on all animals.

### 2.6. Statistical Analyses

Individuals were classified as positive or negative for specific agents based on the results of bacterial culture, viral serology, and fecal parasitology. For data collected multiple times from an individual, data from the first sampling event was used, except for temporal analyses. 

Statistical analysis was conducted using SAS 9.2 (SAS Institute, Inc., Cary, NC, USA). Chi-square analyses were conducted using the FREQ procedure with the threshold for significance set at *P* ≤ 0.05. Odds ratios (ORs) were calculated for 2 by 2 tables. Chi-square tests assume independence of counts. Separate Chi-square analyses were conducted for each host species' Pasteurellaceae isolates to evaluate associations between the host population's interface status (i.e., whether the host population is at an interface or is isolated) and whether isolates were beta-hemolytic (which often is interpreted as presumptive evidence of pathogenicity).

## 3. Results

### 3.1. Populations Studied

Population sizes for sampled bighorn and domestic sheep varied (Table 1). Bighorn sheep primarily inhabited public land, whereas domestic sheep primarily resided on private land for operations managed for wool, meat, or mixed objectives. Bighorn sheep were 1–14 y, and domestic sheep were 1–10 y. Domestic sheep with evidence of mild respiratory disease were found in four interface populations (*n* = 10) and one isolated population (*n* = 1). No domestic goats or bighorn sheep had evidence of respiratory disease. 

### 3.2. Bacteriology: Pasteurellaceae

Two hundred sixty-five bighorn sheep, 203 domestic goat, and 790 domestic sheep Pasteurellaceae isolates were identified (Table 2). These isolates comprised 166 unique Pasteurellaceae species or biovariants that were often identified in multiple host species. Bighorn sheep, domestic goats, and domestic sheep had 60, 37, and 135 different Pasteurellaceae species or biovariants isolated, respectively. Thirty six of the bighorn sheep Pasteurellaceae species or biovariants were also found in domestic livestock, and this overlap represented 72% (*n* = 190) of the bighorn sheep, 82% (*n* = 167) of domestic goat, and 58% (*n* = 462) of domestic sheep Pasteurellaceae isolates. Bighorn sheep isolates were primarily (73%) *P*. (*B*.) *trehalosi* (*n* = 193), whereas most (60%) of domestic sheep isolates were *M*. *haemolytica* (*n* = 473). Half (50%) of domestic goat isolates were *P*. (*B*.) *trehalosi* (*n* = 102), and 44% were *M*. *haemolytica* (*n* = 89). There were also 375 bighorn sheep, 96 domestic goat, and 448 domestic sheep isolates that were not characterized as Pasteurellaceae, including *Arcanobacterium pyogenes*, *Bacillus* sp., *Enterobacter *sp., *Enterococcus* sp., *Moraxella* sp., *Neisseria* sp., *Proteus* sp., *Pseudomonas* sp., *Staphylococcus* sp., *Streptococcus* sp., and coliforms. 

Twenty-two biovariants were found in interface bighorn sheep populations but not in isolated populations. For comparison, 24 biovariants were found in isolated bighorn sheep populations but not in interface populations. Among domestic sheep, 38 biovariants were found only in interface populations, and 52 biovariants were found only in isolated populations. There was not a significant association between whether a Pasteurellaceae isolate was beta-hemolytic and whether the isolate was collected at the interface for domestic (*P* = 0.89; OR 0.98, 95% CI 0.71–1.34) or bighorn sheep populations (*P* = 0.41; OR 0.76, 95% CI 0.40–1.45).

Individual domestic sheep (*n* = 85) and goats (*n* = 34) from three sheep and one goat operation were resampled 6 mo apart to assess temporal variation in Pasteurellaceae biovariants. None of the domestic sheep and goats sampled twice had complete concordance in the biovariants identified for each sampling period. Among the domestic sheep (*n* = 493) and goat (*n* = 219) isolates that were identified in both sampling periods, only 4% of sheep (*n* = 20) and goat (*n* = 9) isolates were identified at both sample events from the same individual. Two isolates were identified in the same domestic sheep during both sample periods on two occasions. 

### 3.3. Bacteriology: *Mycoplasma*

Swabs from bighorn sheep (*n* = 248), domestic sheep (*n* = 166), and domestic goat (*n* = 18) were cultured for *Mycoplasma*. *Mycoplasma* was isolated from 60–100% of sampled individuals in each of the domestic livestock populations, with the exception of one domestic sheep population without *Mycoplasma* isolates from 13 sampled animals. In contrast, *Mycoplasma* was not isolated from any of the bighorn sheep sampled. 

### 3.4. Bacteriology: Euthanized Bighorn Sheep

A male bighorn sheep euthanized for closely associated with domestic sheep and goats had no apparent clinical disease. Two biovariants *P*. (*B*.) *trehalosi *2^CDS^ and *P*. (*B*.) *trehalosi *4^CDS^) isolated from this male were not identified in the closest bighorn sheep population, but were identified in the sympatric domestic livestock. *Pasteurella* (*B*.) *trehalosi *2^B^, which was the most common biovariant isolated from bighorn sheep (*n* = 88), was isolated from the euthanized male and was identified in one sympatric domestic sheep. *Pasteurella* (*B*.) *trehalosi* 2, *Bacillus* sp., and *Arcanobacterium pyogenes* were also isolated from the bighorn sheep male and sympatric livestock. Samples from this male were negative for *Mycoplasma* sp., although *Mycoplasma* sp. was isolated from the sympatric goat and domestic sheep population.

### 3.5. Virology

Every population tested had serologic evidence of PI-3 virus (Table 3). All populations except for some isolated populations of domestic sheep (*n* = 1) and bighorn sheep (*n* = 3) had serologic evidence for BRSV. Five individuals in two domestic sheep populations had serologic evidence for both BVDV-1 and BVDV-2, and all titers were <1 : 128. Two bighorn sheep and one goat had low titers (1 : 8) to IBR, and the goat was at the same interface as one of the bighorn sheep. Of the domestic sheep (*n* = 85) in three populations and domestic goats (*n* = 34) in one population that were sampled six months apart, there was evidence for seroconversion to PI-3 (*n* = 26) and BRSV (*n* = 5). For domestic sheep with signs of respiratory disease (*n* = 11), there was evidence for antibodies to PI-3 (*n* = 9) and BRSV (*n* = 5), but not BVDV-1, BVDV-2, or IBR.

### 3.6. Parasitology

Fecal samples were evaluated for isolated (*n* = 165) and interface (*n* = 98) bighorn sheep among six isolated and three interface populations. Fecal samples were evaluated for isolated (*n* = 36) and interface (*n* = 44) domestic sheep among three isolated and six interface populations. Twelve fecal samples were analyzed from the goat population. *Protostrongylus* and *Dictyocaulus* sp. were identified in isolated and interface bighorn sheep populations, whereas dorsal-spined larvae presumed to be *Muellerius* sp. were identified in an isolated bighorn sheep population. Representatives of Trichostongylina, *Moniezia* spp., *Strongyloides* sp., and *Eimeria* sp. were identified in the goat population. Trichostongylina, *Moniezia* spp., and *Eimeria* sp. were identified in isolated and interface domestic sheep populations, whereas *Strongyloides* sp. and *Dictyocaulus *sp. were identified in interface domestic sheep populations. *Muellerius* sp. was not identified in domestic goat and sheep populations. Livestock were treated with anthelmintics at least once during the previous year.

## 4. Discussion

This study documented the presence of multiple bacterial, viral, and parasite species in bighorn sheep and livestock populations distant from and at the domestic animal/wildlife interface. Although the sites where these populations were sampled do not reflect the extensive translocations of bighorn sheep that have been conducted for management purposes or individual animal movements between populations [3], these results provide important baseline data for understanding agents potentially responsible for respiratory disease in bighorn and domestic sheep under field conditions. Populations of varying size (Table 1) were sampled opportunistically based on agency or collaborator activities with bighorn sheep, and livestock operator's willingness to participate. Consequently, based on standards for observational studies [41], the limitations of extrapolating inferences from this study to other populations and locations must be acknowledged. Also, few males were sampled in this study, and all wildlife and domestic populations at the interfaces studied could not be sampled. 

### 4.1. Populations Studied

Sampled bighorn sheep populations varied by an order of magnitude in estimated size and density (Table 1). Domestic sheep populations varied by two orders of magnitude and ranged from small family operations to large, open range populations. While bighorn sheep were exclusively on public land (federal and state), domestic livestock were largely on private land. This finding is, in part, a function of the populations sampled for this study. However, it illustrates that, while there has been contention over domestic livestock grazing on public lands [42, 43], the potential for conflicting management objectives also exists where domestic livestock are on private land near to public lands that are populated with bighorn sheep. The range in sizes of sampled bighorn and domestic sheep populations met the objective of establishing representative baseline data, although further research is needed to establish whether there are patterns associated with population size.

### 4.2. Bacteriology: Pasteurellaceae

Pasteurellaceae are a heterogeneous mix of many bacterial strains that can cause a range of clinical signs [44]. For this study, an established biovariant classification scheme was used for Pasteurellaceae isolates because it offered the best opportunity to classify isolates and provide useful epidemiological data. Collection of swabs for bacteriological cultures was conducted according to standardized sampling and laboratory procedures developed by the Western Wildlife Health Committee, Association of Western Fish and Wildlife Agencies [28].

Pasteurellaceae associated with respiratory disease in bighorn and domestic sheep are *M. *(*Pasteurella*) *haemolytica*, *P*. (*B.*) *trehalosi* (formerly *P*. *haemolytica* biotype T), and *P*. *multocida* [44–50]. These bacteria are also found in animals without disease, whether serotype or biovariant subclassification schemes are used to characterize isolates [33, 51–54]. Under in vivo and in vitro experimental conditions, bighorn sheep appear to be more susceptible to disease due to *M. haemolytica* than are domestic sheep and other species [17, 18, 55, 56]. It is uncertain whether a similar etiopathogenesis occurs with other Pasteurallaceae species and how experimental results can be applied to predicting and mitigating respiratory disease outbreaks in free-ranging populations. 

Multiple Pasteurellaceae species or biovariants were identified in the host species in this study, and most biovariants were represented by only a few isolates (Table 2). While *M. haemolytica* and *P*. *multocida* were primarily associated with domestic sheep and *P*. (*B*.) *trehalosi* comprised most bighorn sheep isolates, Pasteurellaceae biovariants often occurred among multiple host species, and there was a complex assemblage of Pasteurellaceae and non-Pasteurellaceae species [57]. This is similar to results of previous studies in Nevada and California that used biovariant and serotype classification schemes, and it presents challenges for identifying patterns and making rigorous inferences [53, 54]. These challenges are expanded by the overlap of isolates identified in this study among largely healthy animals compared with retrospective studies of domestic and bighorn sheep with respiratory disease [58, 59]. 

The cross-sectional study design and the dearth of animals with clinical signs of disease preclude identification of pathogenic Pasteurellaceae or the potential for cross-species transmission to result in disease, but data is presented that is germane to these topics. There was no evidence for an increased risk for beta-hemolytic or “unique” biovariants to be identified in interface populations. This suggests that bighorn and domestic sheep may be colonized by leukotoxin-positive Pasteurellaceae without the development of disease, although further work is needed to clarify whether the numerous biovariants and their occurrence in multiple host species could easily obscure cross-species transmission of any Pasteurellaceae that might have occurred. Similarly, the observation of a single, apparently healthy bighorn sheep ram that shared shelter, food, and water with domestic sheep and goats for >6 mo has limited inference regarding cross-species transmission in either direction. However, the identification of biovariants that were not found in other members of the same species in the ram and one domestic sheep is consistent with, but not definitive for, cross-species transmission. Further study using DNA fingerprinting or sequencing technology would be needed to confirm the similarity of these isolates but would not confirm direction of transmission without longitudinal data. Nevertheless, these data support removal of individuals that associate with sympatric species where there is a low tolerance for possible interspecies transmission of agents. 

Pasteurellaceae results from individual livestock resampled 6 mo apart were compared as a means of assessing temporal variation (Table 3). Minimal concordance in culture results for these individuals suggests that oropharyngeal microflora may be temporally dynamic, that sample numbers or swabbing methods may be inadequate to characterize the great diversity of biovariants that are present, or that competition among biovariants in culture or standard microbial culture procedures are responsible for this variation. Regardless of the reasons for these results, single sampling events for domestic livestock may not be appropriate for some research and management questions. A previous study similarly suggests that bighorn sheep Pasteurellaceae may vary temporally, but results were confounded by use of antibiotics [34]. It is unlikely that this variation is due to inconsistent classification of isolates, because there is substantial consistency in assigned Pasteurellaceae biovariant classifications among isolates that are recharacterized as a part of routine reference bank and quality control procedures.

The diversity of Pasteurellaceae observed in this study and others presents challenges for interpretation. The observation of many, uncommonly identified biovariants in this study indicate that more extensive sampling is required to fully characterize the Pasteurellaceae of bighorn sheep and sympatric livestock. Additional data from animals with respiratory disease is needed to determine the pathogenicity of biovariants, as well as the impact upon populations. Temporal variation or inconsistent detection of Pasteurellaceae suggests the need to develop population level sampling strategies and interpretations of agent health impacts [60]. Although there has been extensive research on Pasteurellaceae biovariants and their pathogenesis in bighorn sheep, future work that characterizes risk factors for individuals and that compares populations may yield data that is useful for management purposes. 

### 4.3. Bacteriology: *Mycoplasma* sp.

*Mycoplasma ovipneumoniae* has been associated with respiratory disease as a primary pathogen of small ruminants and may increase their susceptibility to secondary pasteurellosis, particularly in lambs 2–12 months of age [61, 62]. This could explain reduced lamb recruitment following bighorn sheep outbreaks [48, 62, 63]. However, undifferentiated *Mycoplasma* was isolated from apparently healthy animals in all but one domestic livestock population in this study. This is similar to previous studies that suggest that *M*. *ovipneumoniae* and possibly other *Mycoplasma* species may be common respiratory tract commensals that only cause disease in animals that are compromised due to other causes [64]. 

In contrast to domestic sheep, bighorn sheep populations in this study appeared to be naïve to *Mycoplasma*. This naivety suggests the potential for an outbreak if this agent was introduced. Alternatively, although the species of *Mycoplasma* was not established, *Mycoplasma* may not be easily transmitted to bighorn sheep. Limited inference to support this is the failure to isolate *Mycoplasma* from the single male bighorn sheep that was closely associated with livestock infected with *Mycoplasma*. 

Whether *Mycoplasma* can be an opportunistic or primary pathogen in free-ranging bighorn sheep is not clear. While a high degree of association between *M*. *ovipneumoniae *and bronchopneumonia in free-ranging bighorn sheep has been reported, the percentage of free-ranging pneumonic bighorn with culture or PCR evidence of *Mycoplasma* infection varies from 7 to 55% [23, 36, 48]. In addition, limited experimental inoculations in bighorn sheep lambs suggest that *M*. *ovipneumoniae* infection alone is insufficient to cause fatal respiratory disease [23]. This variation could be due to methodological limitations [23], variation in the agents responsible for different outbreaks, or other reasons. In particular, it is important to recognize that *Mycoplasma* sp. is not routinely isolated with general microbiological methods that are routinely employed by diagnostic laboratories. While this study employed methods developed for *Mycoplasma *sp. by a laboratory with expertise in *Mycoplasma* sp. isolation, there is a need for additional research to clarify methods and sample sizes that are required for strong inferences from research on bighorn and domestic sheep populations.

### 4.4. Virology

The viral respiratory agents in this study were selected based on their potential to cause respiratory disease or predispose to pneumonic pasteurellosis in domestic and wild ruminants [22, 65–67]. A high percentage of the domestic livestock and bighorn sheep in this study had evidence of antibodies to PI-3 and BRSV (Table 3). There was evidence of seroconversion for BRSV and PI-3 among domestic sheep and goats that were sampled twice. Parainfluenza-3 and BRSV (or reported as RSV) have been associated with respiratory disease in bighorn sheep, and domestic sheep and goats [36, 65, 68, 69]. However, antibodies to these agents were also present in apparently healthy animals in these references and others [22, 36, 70–72]. This indicates that survival from infections is possible and perhaps probable in populations with high serologic prevalence.

In contrast to PI-3 and BRSV, few animals had evidence of antibodies to BVDV-1, BVDV-2, or IBR (Table 3). Limited documentation exists on the clinical effect of these infections in domestic sheep and goats [68, 69, 73]. Serologic evidence of BVDV and IBR infections in healthy bighorn sheep indicates that these animals can survive infections with these agents [22, 70]. However, isolation of IBR from 3 of 6 lung samples from bighorn sheep during a Tendoys, Montana outbreak, isolation of BVDV from 14 of 19 bighorn sheep lungs during a Lost Creek, Montana outbreak [22], and > fourfold increases in serologic titers to BVDV during the Hells Canyon outbreak [36] suggest a potential role for these viruses in some bighorn sheep die-offs. Based on domestic ruminant models, these viruses may cause primary infections that result in secondary, opportunistic pneumonic pasteurellosis under some conditions or be non- to mildly pathogenic in other circumstances [36, 65, 74]. Further research is needed to clarify the degree, frequency, and circumstances under which these agents pose a risk for causing disease.

### 4.5. Parasitology

Evaluations of parasitic infections based on fecal analyses (helminth larvae and eggs, oocysts) are included in this study due to the potential for gastrointestinal parasites to predispose animals to disease from other agents and the role of lungworms in ungulate respiratory disease [12, 67, 75]. Only presence and absence data are reported herein as validated and standardized, and quantitative methods for assessing parasite numbers were not available for this study. The nematode, cestode, and protozoan parasites identified are similar to those previously reported for domestic and bighorn sheep, and their impact on the populations studied are uncertain [75–77]. *Muellerius* sp. is generally associated with domestic sheep, rather than bighorn sheep [78]. Evidence for *Muellerius* sp. in an isolated bighorn sheep population may represent historic introduction and establishment of this parasite in this bighorn sheep population, or unidentified recent introduction [79]. Additionally, it has become apparent that not all reports of dorsal-spined larvae may be attributable to *Muellerius* but could represent another protostrongylid muscleworm, *Parelaphostrongylus odocoilei* known to infect bighorn at some localities [79, 80]. Across this assemblage of free-ranging and domestic host species, historic introductions in other settings are thought to be responsible for a mosaic landscape of native and introduced parasite species [81]. This suggests the potential for a similar mosaic faunal structure for bacterial and viral agents, as a consequence of historic transmission events across the bighorn/domestic sheep interface, which could account from some of the results in this study. 

## 5. Conclusions

This study documents the presence of multiple Pasteurellaceae biovariants, *Mycoplasma*, and viruses in apparently healthy bighorn sheep, domestic sheep, and goats that are at the domestic animal/wildlife interface and in isolated populations. When these results are considered with their presence in animals with respiratory disease in other reports [22, 23, 36, 58, 59], it is evident that further work is needed to clarify environmental, agent, and host determinants of respiratory disease, as well as standardize sampling and laboratory procedures. Clarification of whether these agents are primary pathogens, secondary pathogens, commensals, or predispose to outbreaks due to other agents is important. 

Given the polarized debate over management practices at the bighorn/domestic sheep interface, the potential for results of this study to be selectively interpreted exists. Reflecting upon basic animal disease control principles and how they might be applied to free-ranging wildlife will be more useful. Any time contact occurs among populations, the potential for transmission of novel agents to naïve animals exists [82], and there are established quarantine, vaccination, testing, risk assessment, and other strategies for minimizing spread of infectious diseases among translocated domestic and wild animal populations [83–85]. However, there is a need to rigorously document the true risks of interspecies transmission under field conditions, as well as determine the efficacy of different management strategies. Strategies may vary by circumstances, as management of small or otherwise highly valued bighorn sheep populations may result in adoption of risk-averse strategies. Similarly, domestic sheep operations that are considered critical for a local economy, for exotic weed control, to prevent conversion of land to uses that are not compatible with wildlife or agricultural interests, or for other reasons, may require management strategies that protect their interests. For all other situations, management will be guided by sociological values and biological perceptions until the biological risks and options are clarified, and a sociologically based structure for decision making is agreed upon.

## Figures and Tables

**Table 1:** Characteristics of bighorn sheep and domestic sheep populations studied, based on proximity to the bighorn/domestic sheep interface.

		Bighorn sheep^1^		Domestic goats^2^	Domestic sheep^2^	
		Isolated populations^3^	Interface populations^3^	Interface populations^3^	Interface populations^3^	Isolated populations^3^
	No. of populations:	7	3	1	6	6
Population size	Median	313	175	925^4^	105^4^	510^4^
	Min.–max.	35–750	70–350	—	25–1780	30–4000
Population density (No./km)	Min.–max.	0.3–1.9	0.3–0.7	—	—	—
No. of animals sampled	Total	234	106	45	152	219
	Min.–max per population	6–81	26–49	—	19–70	20–70
Land occupied-	Public^5^	7	3^6^	0	0	2
winter	Private	0	0^6^	1	6	4
Land occupied-	Public^5^	7	3^6^	0^7^	1^7^	3
summer	Private	0	0^6^	1^7^	6^7^	3

^1^Data based on 2003 annual aerial census by Montana Fish, Wildlife and Parks.

^2^Data from questionnaire.

^3^Based on 14.5 km barrier recommended for land management [25]; interface ≤14.5 km, relative to sympatric species, and isolated >14.5 km, relative to sympatric species (or surrounded by development that prevents interactions with sympatric species).

^4^Number of females in population.

^5^Public land: federal and state lands.

^6^One population 50% federal and 50% private land.

^7^One population 10% on public land.

**Table 2:** Pasteurellaceae biovariants and subspecies (no. of isolates) cultured from individual isolated and interface bighorn sheep (*n* = 10 populations), domestic sheep (*n* = 12 populations), and goats (*n* = 1 population).

Host species:	Bighorn sheep		Goat	Domestic sheep	
Interface^1^	No	Yes	Yes	Yes	No
No. of populations	7	3	1	6	6
No. of animals:	234	105	45	152	219
No. of Pasteurellaceae isolates	144	121	203	389	401
Species					

*Actinobacillus*	1		6	35	16
*Mannheimia haemolytica*	1(1), 1^*α*^ (2)	1(1), 1^*α*^ (1), 1^G^ (2)	1 (18), 1^*α*^ (2),	1 (21), 1^*α*^ (4),	1 (28), 1^*α*^ (4),
			1^*α*B^ (1), 1^*α*G^ (1),	1^*α*B^ (1), 1^*α*BG^ (2),	1^*α*B^ (1), 1^*α*E^ (1),
			1^B^ (1), 1^E^ (10),	1^*α*G^ (1), 1^EG^ (1),	1^*α*G^ (1), 1^E^ (1),
			1^EG^ (1), 1^G^ (5)	1^G^ (11)	1^EG^ (1), 1^G^ (8)
	3^*α*BCE^ (1),	3^*α*CD^ (1),	3 (3), 3^*β*^ (1),	3 (17), 3^*α*^ (1),	3 (18), 3^*α*^ (1),
	3^*α*BG^ (1), B (2)	3^CDE^ (2), 3^G^ (1)	3^*β*BDE^ (1), 3^C^ (1)	3^*α*G^ (1), 3^*β*^ (2),	3^*α*BDE^ (1), 3^*α*BDE^ (1),
				3^*β*CDE^ (1), 3^C^ (1),	3^*α*BDE^ (1), 3^*β*CD^ (1),
				3^CD^ (1), 3^CE^ (1),	3^*β*D^ (2), 3^D^ (1),
				3^DE^ (1), 3^E^ (5)	3^E^ (1)
				5 (9), 5^*α*^ (2),	5 (12), 5^*α*B^ (2),
				5^*α*B^ (2), 5^*αβ*G^ (1),	5^*αβ*BD^ (1), 5^B^ (4),
				5^C^ (1)	5^*β*BD^ (2), 5^*β*B^ (1),
					5^D^ (1)
	6^*α*R^ (1)	6^*α*R^ (1)			6^*α*R^ (2)
			7 (3), 7^X^ (2)	7 (3), 7^B^ (1),	7 (4), 7^B^ (6),
				7^BX^ (1), 7^X^ (5)	7^BGX^7 (1), 7^BX^ (3),
					7^X^ (3)
	8 (1)	8 (1), 8^B^ (1)	8 (6)	8 (7), 8^B^ (6)	8 (19), 8^B^ (3),
					8^G^ (2)
	9^*αβ*B^ (1)	9^*α*^ (1), 9^*αβ*B^ (1),	9^R^ (1)	9^*αβ*^ (1)	9 (1), 9^*α*B^ (1),
		9^*αβ*R^ (3)			9^*αβ*^ (1), 9^*αβ*B^ (1),
					9^*β*B^ (2)
	10 (5), 10^*α*^ (3),	10^*α*BE^ (1), 10^B^ (2)	10^C^ (1)	10 (3), 10^*α*^ (3),	10 (13), 10^*α*^ (8),
	10^*α*BCG^ (1), 10^*α*BE^ (1)			10^*α*C ^(1)	10^C^ (1)
	11 (2), 11^*α*BCE^ (1),	11^E^ (7)	11^E^ (1)	11 (26), 11^*α*^ (5),	11 (22), 11^*α*^ (1),
	11^*α*DEGX^ (1), 11^BC^ (1)			11^*α*E^ (1), 11^*α*EC^ (1),	11^*α*BEX^ (1), 11^*α*C^ (1),
				11^*β*^ (6), 11^BC^ (1),	11^E^ (7)
				11^E^ (10)	
	16^*α*^ (1),	16^*α*BEG^ (1)	16^*α*^ (4), 16^*α*E^ (2)	16^*α*BE^ (3), 16^*α*D^ (1),	16^*α*^ (2), 16^*α*B^ (1),
	16^*α*BEG^ (1)			16^*α*E^ (6), 16^*α*EG^ (2),	16^*α*BE^ (5), 16^*α*BG^ (1),
				16^*α*G^ (2), 16^B^ (1),	16^*α*E^ (4), 16^B^ (6),
				16^BE^ (1), 16^G^ (2)	16^BE^ (3), 16^BEG^ (1),
					16^E^ (4), 16^EG^ (1),
					16^G^ (1)
	U^*α*^ (1), U^*α*EX^ (1)	U^*α*B^ (1), U^*α*B^ (1),	U (1), U^*αβ*B^ (1),	U^*αβ*^ (2), U^*αβ*X^ (3),	U^*α*^ (3), U^*αβ*B^ (5),
		U^*αβ*EG^ (2), U^*α*E^ (1),	U^*αβ*^ (5), U^*αβ*G^ (1),	U^*α*CER^ (2), U^*α*EX^ (1),	U^*αβ*BX^ (4), U^*αβ*GX^ (1),
		U^*β*^ (1), U^*β*B^ (1),	U^*β*^ (14), U^*β*R^ (1),	U^*α*ERX^ (1), U^*β*BE^ (1),	U^*αβ*^ (11), U^*αβ*G^ (1),
		U^*β*E^ (1)	U^R^ (1)	U^*β*B^ (1), U^*β*BX^ (2),	U^*αβ*X^ (3), U^*α*E^ (2),
				U^*β*G^ (1), U^BEX^ (1),	U^*α*X^ (1), U^*β*^ (2),
				U^E^ (1)	U^*β*BEX^ (4), U^*β*BX^ (1),
					U^*β*EX^ (1), U^B^ (1),
					U^BE^ (1), U^EX^ (2)

*Pasteurella multocida*	*multocida* a (1)	*multocida* a (4),	*multocida* a (2),	*multocida* a (7),	*multocida* a (1),
		biotype U^6^ (2)	biotype U^16^ (1),	*multocida* b (7),	*septica* (10),
			biotype U^6^ (3)	*canis* (4),	biotype U^23^ (1),
				*Septica* (35),	biotype U^24^ (1)
				biotype U^6^ (3)	
*Pasteurella (Bibersteinia) trehalosi*	2 (32), 2^B^ (53),	2^B^ (35), 2^BCDE^ (1),	2 (25), 2^C^ (7),	2 (53), 2^B^ (1),	2 (63), 2^BCDE^ (1),
	2^BC^ (1), 2^BCE^ (1),	2^BD^ (4), 2^BE^ (11),	2^CD^ (1), 2^CDS^ (1),	2^C^ (2), 2^CDES^ (2),	2^C^ (7), 2^CDE^ (1),
	2^BD^ (1), 2^BE^ (12),	2^BG^ (4), 2^BS^ (13),	2^CE^ (1), 2^E^ (62)	2^CDS^ (2), 2^CE^ (1),	2^CDES^ (2), 2^CDS^ (1),
	2^BGS^ (1), 2^C^ (1),	2^C^ (1), 2^E^ (3),		2^CES^ (1), 2^CS^ (2),	2^CE^ (1), 2^DES^ (1),
	2^CD^ (1), 2^CDS^ (1),	2^G^ (3), 2^GS^ (1),		2^E^ (5), 2^EG^ (1),	2^E^ (2), 2^EG^ (1),
	2^E^ (2), 2^EG^ (1), 2^GS^ (1)	2^S^ (4)		2^EGS^ (1), 2^ES^ (1),	2^G^ (4), 2^S^ (1)
				2^G^ (1), 2^GS^ (1),	
				2^S^ (4)	
	4^B^ (3), 4^BDS^ (1),		4^CDS^ (5)	4 (1), 4^CDES^ (2),	4 (1), 4^BCDS^ (1),
	4^CDS^ (1)			4^CDS^ (14), 4^G^ (1)	4^CD^ (1), 4^CDE^ (3),
					4^CDEGS^ (1), 4^CDES^ (6),
					4^CDS^ (4)

^1^Yes: ≤14.5 km to sympatric species; No: >14.5 km to sympatric species or surrounded by development that prevents interspecific interactions.

**Table 3:** Number (%) of bighorn sheep, domestic sheep, and domestic goats with serologic evidence for antibodies to parainfluenza-3, bovine respiratory syncytial virus, bovine viral diarrhea-1 and bovine viral diarrhea-2, and infectious bovine rhinotracheitis in isolated and interface populations.

Species	Bighorn		Domestic goat	Domestic sheep	
Location	Isolated	Interface	Interface	Interface	Isolated
No. of populations	7	3	1	6	6
No. of animals tested	198	105	44	143	214
Parainfluenza-3	165 (83%)	91 (87%)	9 (20%)	102 (71%)	113 (53%)
Bovine respiratory syncytial virus	57 (29%)	76 (72%)	44 (100%)	95 (66%)	104 (49%)
Bovine viral diarrhea-1	0 (0%)	0 (0%)	0 (0%)	1 (0.7%)	3 (1%)
Bovine viral diarrhea-2	1 (0.5%)	0 (0%)	0 (0%)	1 (0.7%)	6 (3%)
Infectious bovine rhinotracheitis	0 (0%)	2 (2%)	1 (2%)	0 (0%)	0 (0%)
